# Two Cases of Removal of Omental Tumor from the Scrotum

**Published:** 1875-08

**Authors:** J. F. Miner


ART. II.—*Two Cases of Removal of Omental Tumor from the Scrotum.* By Prof. J. F. MINER, M. D. Reported by E. N. BRUSH, M. D.

It is believed that the rarity of cases similar to the two following, and the operative procedures undertaken for their relief, will render a detailed account not uninteresting:

CASE I.—E. T. D., a druggist of Jamestown, N. Y., applied to Dr. J. F. Miner in October, 1873, for relief from what he and the physicians hitherto consulted had supposed to be enlarged testicle. From infancy he had been troubled with an enlargement of the scrotum on the right side which had increased recently as Mr. D. had increased in flesh. The patient was a healthy young man, aged about twenty-eight, weighing two hundred pounds. There was no evidence of any hereditary disorder, the tumor was not painful, and was only troublesome on account of its size and weight. There was no decrease in size in the recumbent position, nor could the growth be returned to the abdominal cavity, though it evidently extended into the inguinal canal.

The patient secured a private room at the Buffalo General Hospital, and made arrangements to have the tumor removed.

Oct. 15th. The patient being placed under ether, opportunity was afforded for a more careful examination than had hitherto been afforded. By careful manipulation the testicle, of normal size and apparently healthy, could be isolated from the growth, but the exact character of the tumor could not be diagnosed.

Drs. Hazeltine and Barnes, who were present, concurring in the propriety of the procedure, Dr. Miner carefully cut through the coverings of the tumor, making an incision about four inches in length, in line with the inguinal canal. After the walls of the scrotum were divided, a thin transparent sac was discovered (afterwards found to be a reduplication of peritoneum) containing what was apparently a fatty tumor of considerable size. This sack was opened and an effort made to remove the tumor. It was then discovered that it was a protrusion of omentum which had probably descended with the testicle in infancy, and had increased in size as the patient had grown fleshy. Following the protrusion up the inguinal canal it was found that it was firmly adherent on all sides to the lower portion of the canal. The cord and testicle were found in a healthy condition. The mass of omentum had become unfolded to such an extent that it was impossible to return it within the scrotum, and the adhesions precluded the possibility, if desirable, of returning it to the abdominal cavity. Nothing remained, therefore, but to cut it away. A stout ligature was thrown around the mass at the lower end of the canal and the omentum cut away with scissors. The ligature controlled all hemorrhage and no vessels were ligated.

The incision was closed, leaving the lower angle open for drainage, warm water dressings applied and the patient placed in bed.

On recovering from the anæsthetic the patient complained of considerable pain, and one-fourth grain morphia was given hypodermically.

Oct. 16th. Some pain complained of during the night—pulse is 120.

17th. It was found necessary to draw the urine. Pulse 120. Anodynes continued to relieve pain.

18th. Pulse 114. Less fever, skin moist, some discharge from the wound.

20th. Scrotum swollen and painful; a free incision at lower portion gives exit to considerable pus and some debris of tissue. No abdominal tenderness.

21st. Pulse 96. Restless and imaginary. Sutures removed. The wound is united in the greater portion of its extent.

22d and 23d. Delerious. Pulse 120 to 130. Temp. 103°. Is given chloral hydrate which produces some sleep.

24th. Improving. From this date until his discharge the patient continued to improve, and on the 9th of Nov., he left for his home

The mass of omentum removed weighed two and one-half pounds. Its dimensions were not taken, but when unfolded it covered a large space.

Case II.—J. B——, of Mt. Morris, was referred to Dr. Miner by Dr. W. W. Potter of that place. The patient is sixty-five years of age, in apparent good health, and weighed three hundred and twenty pounds. Has increased in flesh to a large amount during the last eight years.

He had never had hernia, and previous to his increase in flesh never noticed any growth in his scrotum. Eight years ago he began to notice an enlargement in the right side of the scrotum, which was at first pronounced varicocele, but no pallitive treatment was undertaken.

This growth gradually enlarged until its size and weight became oppressive, and Mr. B. consulted several physicians in regard to it, but no one gave a decided opinion as to its nature. A prominent surgeon in Boston declined to give an opinion as to its nature. Two surgeons in New York State were consulted but advised no treatment except a suspensory bandage.

When he consulted Dr. Miner, the tumor had attained such a size that the penis was entirely obliterated, the preputial orifice alone being seen at the top of the tumor. The tumor had a peculiar feeling, which gave almost the sensation of fluctuation, and to assist in the diagnosis a small exploring trocar was introduced. Nothing escaped from the trocar except a few drops of serous fluid and a small amount of oily matter. This, together with the feeling of the tumor, convinced Dr. Miner that an opinion which he had previously formed was correct, that it was enlarged omentum. Upon being told the diagnosis made, Mr. B., who was a man of intelligence, and understood the nature of the case, asked if it could be removed. He was answered in the affirmative, and the risks of the operation fully explained to him.

He at once decided upon an operation and the following day was appointed as the time.

July 21st.—The patient having been placed under the influence of ether, Dr. Miner removed the tumor in the presence of Drs. Bartow, W. W. Miner, and the writer. The mass was found inclosed in a peritoneal envelope as in the first case, and firmly adherent to the margins of the inguinal canal. After satisfying himself that no intestine was included in the mass, a ligature was carried around it as high up as the adhesions would allow, and the tumor cut away with scissors.

Upon examination of the mass a concretion was found imbeded in its folds the size of a walnut of a hard cartilaginous nature. Mr. B. had called attention to this previous to the operation and said that it first made its appearance when straining at stool one day, accompanied by slight pain, which soon passed away. The omentum removed did not differ from that seen in fleshy persons in the natural position, it was several inches in width and length and weighed a trifle under three and one-half pounds.

Mr. B. did well until the twenty-third, when he seemed stupid but was easily aroused and answered questions in a clear manner. On the 24th the stupor had increased and was accompanied by evident paralysis of the lower limbs. The urine had to be drawn and the patient assisted whenever he wished to move.

It may here be stated that some weeks previous to the operation Mr. B. had been paralyzed on his left side, from this he had however, apparently nearly recovered.

On the 25th pain and tympanitis appeared in the abdominal region, which gradually increased with nausea and vomiting, and on Monday the 26th, the patient died of peritonitis. It is however safe to say that this result would not have followed had the patient been a younger man, and in a better condition to stand the operation. It was hoped that the adhesions at the abdominal ring would prevent the inflammation from extending in the abdominal cavity. It is frequently the case that large portions of omentum have to be removed in operations for hernia, but I am not aware of the report of a case similar to the two preceding ones. That so large a mass of omentum should form in the scrotum seems at first a little remarkable, but from the nature of the two cases, their tendency to obesity, it could be surmised was it known that a portion of omentum had descended to form the nucleus for further growth.

The surgery of omental growths is not as yet clearly defined, and the report of these two cases may be of value in elucidating the subject.

